# Latent tuberculosis infection is associated with increased unstimulated levels of interferon-gamma in Lima, Peru

**DOI:** 10.1371/journal.pone.0202191

**Published:** 2018-09-13

**Authors:** Moises A. Huaman, David Henson, Paola L. Rondan, Eduardo Ticona, Gustavo Miranda, Richard J. Kryscio, Raquel Mugruza, Ernesto Aranda, Cesar Ticona, Susan Abarca, Paula Heredia, Andres Aguirre, Timothy R. Sterling, Beth A. Garvy, Carl J. Fichtenbaum

**Affiliations:** 1 Department of Internal Medicine, Division of Infectious Diseases, University of Cincinnati College of Medicine, Ohio, United States of America; 2 Department of Medicine, Division of Infectious Diseases, University of Kentucky College of Medicine, Lexington, Kentucky, United States of America; 3 Department of Infectious Diseases and Tropical Medicine, Hospital Nacional Dos de Mayo, Lima, Peru; 4 Department of Internal Medicine, Universidad Nacional Mayor de San Marcos, Lima, Peru; 5 Hospital Nacional Edgardo Rebagliati Martins, Lima, Peru; 6 Departments of Biostatistics and Statistics, University of Kentucky Colleges of Public Health and Arts & Sciences, Lexington, Kentucky, United States of America; 7 Department of Internal Medicine, Division of Infectious Diseases, Wake Forest University School of Medicine, Winston-Salem, North Carolina, United States of America; 8 Universidad Ricardo Palma School of Medicine, Lima, Peru; 9 Sociedad Cientifica de Estudiantes de Medicina Villarealinos, Universidad Nacional Federico Villarreal School of Medicine, Lima, Peru; 10 Department of Medicine, Division of Infectious Diseases, Vanderbilt University School of Medicine, Nashville, Tennessee, United States of America; 11 Department of Microbiology, Immunology and Molecular Genetics, University of Kentucky College of Medicine, Lexington, Kentucky, United States of America; Fundació Institut d’Investigació en Ciències de la Salut Germans Trias i Pujol, Universitat Autònoma de Barcelona, SPAIN

## Abstract

**Background:**

We previously reported increased unstimulated blood levels of interferon-gamma in persons with latent tuberculosis infection (LTBI) in the United States, suggesting enhanced immune activation in LTBI. To investigate this further in a TB-endemic setting, we assessed interferon-gamma levels in persons with and without LTBI in Peru.

**Methods:**

We analyzed data from patients with and without a recent type 1 (spontaneous) acute myocardial infarction (AMI) who were enrolled from two public hospital networks in Lima, Peru, and underwent LTBI testing using the QuantiFERON® TB Gold In-tube (QFT) assay. Participants with a positive QFT test were defined as having LTBI, whereas participants with a negative QFT test were defined as non-LTBI. Unstimulated interferon-gamma was quantified via enzyme-linked immunosorbent assay in the QFT nil-tube, which does not contain antigens. We compared unstimulated interferon-gamma levels between LTBI and non-LTBI groups using the Wilcoxon rank sum test. We used proportional odds modeling for multivariable analysis.

**Results:**

Data from 214 participants were included in this analysis. Of those, 120 (56%) had LTBI. There were no significant differences in age, sex and comorbidities between LTBI and non-LTBI participants, except for recent AMI that was more frequent in LTBI. LTBI participants had higher unstimulated interferon-gamma levels compared to non-LTBI participants (median, interquartile range; 14 pg/mL, 6.5–52.8 vs. 6.5 pg/mL, 4.5–15; *P*<0.01). LTBI remained associated with higher unstimulated interferon-gamma levels after controlling for age, sex, recent AMI, history of hypertension, diabetes mellitus, dyslipidemia, end stage renal disease, malignancy, obesity, and tobacco use (adjusted odds ratio, 2.93; 95% confidence interval, 1.8–4.9). In a sensitivity analysis that excluded participants with AMI, the association between unstimulated interferon-gamma and LTBI remained present (adjusted odds ratio; 3.93; 95% confidence interval, 1.9–8.2).

**Conclusions:**

LTBI was associated with higher unstimulated interferon-gamma levels. These data suggest ongoing immune activation in LTBI.

## Introduction

Latent tuberculosis infection (LTBI) affects approximately one fourth of the world population [1]. It is estimated that 5 to 10% of persons with LTBI will progress to active TB disease during their lifetime [2–4]. The other 90 to 95% will remain “latently” infected. Individuals considered at high risk for TB disease progression should receive LTBI therapy based on current screening and treatment guidelines [5, 6].

The classic model of LTBI proposed that mycobacteria contained within infected granulomas remain dormant. Recent research shows that LTBI is not a completely quiescent state, with wide heterogeneity in mycobacterial metabolic activity and host immune responses within each granuloma [7, 8]. Animal models provide evidence that subsets of mycobacteria undergo replication in chronic *Mycobacterium tuberculosis* infection [9]. Clinically, the activity of mycobacteria in LTBI is suggested by the effectiveness of isoniazid in preventing progression to TB disease, as isoniazid acts on mycolic acid cell wall synthesis and is primarily effective against dividing organisms [10–12]. LTBI has been associated with increased expression of pro-inflammatory mediators, suggesting persistent immune activation related to unresolved infection [13, 14].

Using data from the U.S. National Health Nutritional Examination Survey (NHANES), we previously reported an association between LTBI and elevated unstimulated blood levels of interferon-gamma (IFN-γ), a central cytokine in monocyte/macrophage activation usually produced by activated T cells and natural killer cells [15]. To further assess this finding, we performed a secondary analysis of IFN-γ levels in participants of a cardiovascular disease study conducted in Peru, who underwent LTBI testing via the QuantiFERON® TB Gold-In tube (QFT) assay [16]. By studying this population, we sought to examine IFN-γ levels in a setting where *M*. *tuberculosis* infection is endemic and therefore chances of re-exposure to the pathogen and interactions with the host immune system may be more noticeable; widespread use of Bacillus Calmette-Guerin (BCG) vaccine in this population also distinguishes it from the U.S. population.

## Materials and methods

The primary study was approved by the institutional review boards of the University of Kentucky and the University of Cincinnati in the US, and the ethical committees of Hospital Nacional Dos de Mayo and Hospital Nacional Edgardo Rebagliati Martins in Peru. All participants provided informed written consent for collection of study data. Participants were originally enrolled in a case-control study of patients with and without a recent type 1 (spontaneous) acute myocardial infarction (AMI) conducted in Hospital Nacional Dos de Mayo and Hospital Nacional Edgardo Rebagliati Martins in Lima, Peru between July of 2015 and March of 2017. Cases were patients who had been diagnosed with their first type 1 AMI within one year of study entry. Controls were patients recruited from these same hospital networks who did not have a history of AMI, stroke or peripheral vascular disease. Cases and controls were required not to have clinical evidence of active TB disease at time of study entry. Persons with history of human immunodeficiency virus (HIV) infection, TB disease, and/or LTBI treatment were excluded. A detailed description of the recruiting parameters were previously published [16]. Participants’ demographic information and medical history were collected by self-report. Medical charts were reviewed to confirm accuracy of medical history data provided by participants. QFT testing was performed in all participants by a trained research laboratory technician as instructed by the manufacturer [17]. Briefly, the procedures followed for QFT testing were: 1) 1 mL of blood was collected into each of 3 QFT tubes: a nil tube (negative control tube without antigens), a TB antigen tube (containing the *Mycobacterium tuberculosis* antigens ESAT-6, CFP-10 and TB7.7), and a mitogen tube (positive control tube containing the T cell mitogen phytohemagglutinin); 2) after mixing, the tubes were incubated upright at 37°C as soon as possible and within 16 hours of collection. Following a 16- to 24-hour incubation period, the tubes were centrifuged and plasma was stored frozen until further analysis; 3) the concentration of IFN-γ in each plasma specimen was determined using enzyme-linked immunosorbent assay (ELISA). For interpretation of results, the TB response is calculated as the difference in plasma IFN-γ concentration from the TB antigen tube minus the nil tube. TB responses ≥ 0.35 IU/mL (17.5 pg/mL) and ≥ 25% of nil tube value were considered positive, while TB responses < 0.35 IU/mL (17.5 pg/mL) or TB responses ≥ 0.35 IU/mL (17.5 pg/mL) but < 25% of nil value were considered negative. Indeterminate results were defined as IFN-γ concentrations < 0.50 lU/mL (25 pg/mL) in the mitogen tube minus nil tube, or an IFN-γ value > 8.0 lU/mL (400 pg/mL) in the nil tube [18]. Participants with indeterminate QFT results were excluded from this study. Primary results of the QFT test based on the response to TB antigens were presented previously [16].

We then extracted the IFN-γ values obtained in the QFT nil tubes to examine IFN-γ levels without any *in vitro* antigenic stimulation. Because a non-normal distribution of IFN-γ values was expected based on previous studies, we summarized IFN-γ levels as median and interquartile ranges (IQR) and compared these levels between LTBI and non-LTBI groups using the Wilcoxon rank sum test. For multivariable analysis, we used proportional odds modeling to estimate the association between IFN-γ levels and LTBI, after adjusting for potential confounders. This model assumes the odds of having an IFN-γ value in any quartile higher than the Nth or lower is independent of N = 1, 2, and 3 [19]. We used Stata software (version 12.0) for statistical analyses. *P* values < 0.05 were considered statistically significant. All *P* values were 2-tailed. All participants provided informed written consent for collection of study data.

## Results

Data from 214 participants were included in this analysis. Median age was 62 years (IQR, 56–70), 69% were male, 65% had hypertension, 40% had dyslipidemia, 39% had diabetes mellitus, 30% reported tobacco use, and 23% were obese (body mass index ≥30 kg/m^2^). There were 120 (56%) persons with LTBI and 94 (44%) persons without LTBI based on QFT results. Participant characteristics were not significantly different between the LTBI and non-LTBI groups except for recent AMI (Table 1), which was more frequent in the LTBI group as previously reported [16].

**Table 1:** Characteristics of participants with and without latent tuberculosis infection (LTBI).

Characteristic	LTBI (n = 120)	No LTBI (n = 94)	*P* value
Age in years, median (IQR)	63 (57–68)	61 (55–72)	0.26
Male sex	86 (72%)	62 (66%)	0.37
History of hypertension	76 (63%)	62 (66%)	0.69
History of diabetes mellitus	45 (38%)	38 (40%)	0.66
History of dyslipidemia	49 (41%)	36 (38%)	0.71
End stage renal disease	4 (3%)	2 (2%)	0.60
History of malignancy	6 (5%)	5 (5%)	0.93
Tobacco use	41 (34%)	24 (26%)	0.17
Obesity	25 (21%)	25 (27%)	0.38
Recent acute myocardial infarction	67 (56%)	38 (40%)	0.03

**Fig 1:**
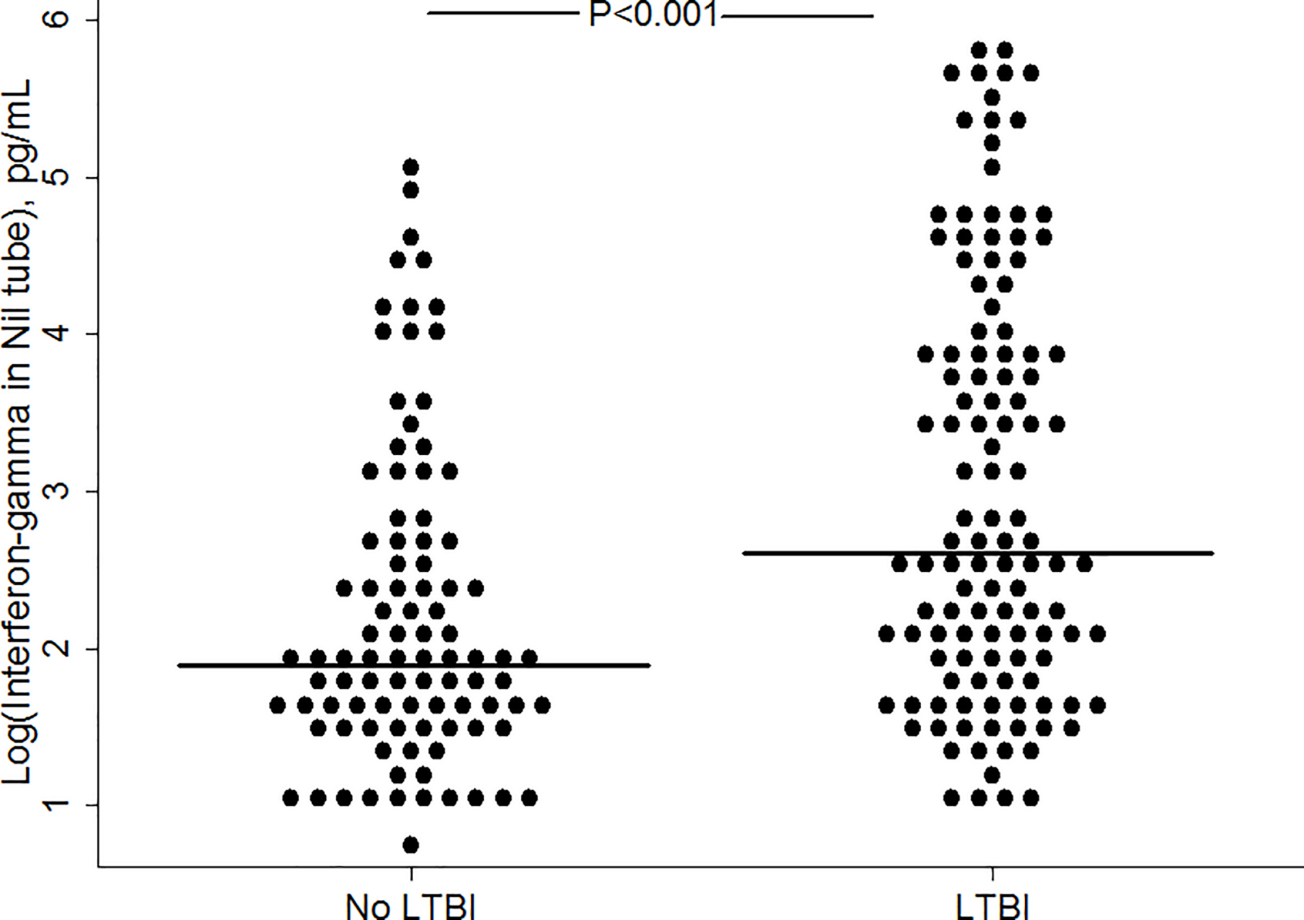
Log of interferon-gamma levels in the unstimulated QuantiFERON® nil tube in persons with and without latent tuberculosis infection (LTBI).

The median values of unstimulated IFN-γ in the QFT nil tube stratified by participant characteristics are shown in Table 2. Unstimulated IFN-γ concentrations were significantly higher in persons with LTBI compared to persons without LTBI (median, IQR; 14 pg/mL, 6.5–52.8 vs. 6.5 pg/mL, 4.5–15; *P*<0.01). The log-transformed IFN-γ levels in the QFT nil tube of persons with and without LTBI are shown in Fig 1. In multivariable analysis, the proportional odds of being in a higher quartile of IFN-γ distribution was almost 3-fold higher for persons with LTBI compared to persons without LTBI, after controlling for age, sex, history of hypertension, diabetes mellitus, dyslipidemia, end stage renal disease, malignancy, tobacco use, obesity and recent AMI (adjusted odds ratio; 2.93, 95% confidence interval, 1.8–4.9). Complete results of the final proportional odds model are shown in Table 3. The proportional odds assumption was not violated (likelihood-ratio test of proportionality of odds, *P* = 0.47; Brant test of parallel regression assumption, *P* = 0.37). In a sensitivity analysis that excluded participants with recent AMI, the association between IFN-γ and LTBI remained present (adjusted odds ratio; 3.93; 95% confidence interval, 1.9–8.2). In a model that only included data from participants with LTBI, we found that none of the participants’ characteristics influenced unstimulated IFN-γ levels within the LTBI population. S1 File contains a de-identified study dataset.

**Table 2:** Interferon-gamma levels in the unstimulated QuantiFERON® nil tube stratified by participant characteristics.

Characteristic	Presence of characteristic		*P* value
	Yes ^a^	No ^a^	
Age above 60 years old	9.5 (5–32.5)	10.5 (6–39.5)	0.76
Male sex	10 (5–35)	9.8 (5.5–32)	0.83
History of hypertension	10 (6–32.5)	9.8 (4.8–46.3)	0.68
History of diabetes mellitus	8.5 (5.5–31.5)	11 (5–41.5)	0.85
History of dyslipidemia	9.5 (5.5–44)	10 (5.5–29.5)	0.59
End stage renal disease	19.8 (6.5–45.5)	10 (5.5–34.5)	0.53
History of malignancy	8.5 (4.5–67)	10 (5.5–34.5)	0.99
Tobacco use	12 (5–86.5)	8.5 (5.5–29)	0.08
Obesity	11.3 (6–36)	9 (5.5–32.5)	0.35
Recent myocardial infarction	11.5 (5.5–41.5)	8.5 (5.5–31.5)	0.16
Latent tuberculosis infection	14 (6.5–52.8)	6.5 (4.5–15)	<0.01

^**a**^ Interferon-gamma values in pg/mL presented as median and interquartile range in parenthesis

**Table 3:** Results of the final proportional odds model for being in a higher quartile of interferon-gamma distribution.

Variable	Adjusted odds ratio (95% CI)
Latent tuberculosis infection	2.93 (1.76–4.9)
Age, per year increase	0.99 (0.96–1.01)
Male sex	0.80 (0.45–1.43)
History of hypertension	1.03 (0.59–1.8)
History of diabetes mellitus	0.95 (0.55–1.64)
History of dyslipidemia	1.09 (0.59–1.8)
End stage renal disease	1.74 (0.63–1.86)
History of malignancy	0.83 (0.24–2.86)
Tobacco use	1.46 (0.8–2.65)
Obesity	1.36 (0.74–2.49)
Recent acute myocardial infarction	1.16 (0.69–1.98)

Because diabetes mellitus induces a dysfunctional immunologic state and is increasingly recognized as a risk factor for developing LTBI and TB disease [20, 21], we conducted a sub-analysis of unstimulated IFN-γ concentrations restricted to the study population with this condition (n = 83). Similar to the results in the overall population, we found that unstimulated IFN-γ levels were higher in diabetic individuals with LTBI compared to no LTBI (median, IQR; 13.5 pg/mL, 6.5–50 vs. 6.8 pg/mL, 4–14; *P*<0.01).

To explore if unstimulated IFN-γ levels in the nil tube influence *M*. *tuberculosis*-specific responses, we assessed the correlation between IFN-γ values in the nil and TB antigen tubes. We found a modest correlation in all subjects (Spearman rho = 0.59; *P*<0.001) and among LTBI individuals (Spearman rho = 0.56; *P*<0.01). We also assessed the correlation between IFN-γ levels in the nil and mitogen tubes. This analysis was limited by a large number of participants with IFN-γ values reported as >10 IU/mL in the mitogen tube (58% of all individuals), who were assigned an absolute value of 10 IU/mL for purposes of this analysis. We found a weak correlation between IFN-γ levels in the nil and mitogen tubes (Spearman rho = 0.22; *P* = 0.01).

## Discussion

We showed that unstimulated blood levels of IFN-γ are higher in persons with LTBI compared to persons without LTBI in a TB-endemic setting such as Lima, Peru. The association between higher IFN-γ levels and LTBI remained present after adjusting for multiple potential confounders. These results are consistent with our previous report of elevated IFN-γ levels in a U.S. population with LTBI [15]. Our results are also consistent with a recent study conducted in Seattle, Washington that found higher geometric mean of QFT nil-tube IFN-γ values in QFT positive vs. QFT negative individuals with and without HIV infection, although these differences were not adjusted by comorbidities [22].

IFN-γ is an important pro-inflammatory cytokine involved in innate and adaptive responses to infection. IFN-γ may be produced by activated lymphocytes, natural killer cells and type 1 innate lymphoid cells [23]. Although the source of IFN-γ in QFT nil tubes is unknown, it may indicate spontaneous IFN-γ secretion by activated lymphocytes and/or other blood cells, thus reflecting a degree of ongoing immune activation in LTBI patients. Supporting this idea, IFN-γ levels in QFT nil tubes are elevated in individuals with other conditions characterized by chronic immune activation such as HIV infection, even when HIV viral loads are < 200 copies/mL [22].

The potential unfavorable consequences of enhanced chronic immune activation in LTBI have been poorly defined. We recently reported an association between LTBI and AMI in this same study population of middle-aged and elderly patients with multiple comorbidities; however, future studies are needed to confirm this finding in other settings [16]. Whether LTBI treatment decreases immune activation requires further investigation, as it could challenge the current paradigm of LTBI management by expanding screening to individuals at increased risk of cardiovascular events and other inflammatory-mediated complications.

Our analysis had limitations. Prior studies showed that unstimulated IFN-γ levels were associated with total peripheral blood lymphocyte counts [15, 22]. We did not measure lymphocyte counts and therefore could not account for this potential confounder. However, since ours consisted of an HIV-negative population and baseline characteristics were similar between the LTBI and non-LTBI groups, unbalanced differences in lymphocyte counts confounding our primary findings are unlikely. Most participants with AMI were recruited within 30 days of their AMI event and therefore IFN-γ concentrations could have been affected by their recent AMI occurrence and/or treatment. To account for this potential confounder, our multivariable analysis adjusted for AMI events and showed an independent association between higher IFN-γ levels and LTBI. Furthermore, in a sensitivity analysis that excluded participants with AMI, the association between IFN-γ and LTBI remained present. Unstimulated IFN-γ levels seemed overall higher than levels reported in prior studies [15, 22], likely because our population was older, primarily inpatient, comorbidities were frequent, and socioeconomic conditions were different across studies. Therefore, these results should not be generalized to the overall population. Nevertheless, our study sample is representative of individuals who may benefit the most from interventions to decrease immune activation. Although having a positive QFT was associated with higher unstimulated IFN-γ responses in our study population, we were unable to investigate whether higher unstimulated IFN-γ levels are associated with an increased (or decreased) risk of progression to TB disease, or whether IFN-γ could be used as a biomarker to target preventive therapy. Prospective studies of baseline unstimulated IFN-γ among persons with and without LTBI, and subsequent TB risk could answer these questions.

In conclusion, our data adds to the growing literature that indicates ongoing immune activation in persons with LTBI. Since LTBI has a wide phenotypic spectrum, studies are needed to identify factors and define LTBI subsets associated with higher immune activation profiles. Similarly, the potential effect of LTBI therapy on immune activation parameters requires further investigation.

## Supporting information

S1 File Dataset. This file contains a de-identified dataset related to the findings described in the manuscript.
(XLSX)
